# Repurposing carvacrol, cinnamaldehyde, and eugenol as potential anti-quorum sensing agents against uropathogenic *Escherichia coli* isolates in Alexandria, Egypt

**DOI:** 10.1186/s12866-023-03055-w

**Published:** 2023-10-23

**Authors:** Hadeer A. Morgaan, Hoda M. G. Omar, Azza S. Zakaria, Nelly M. Mohamed

**Affiliations:** Microbiology and Immunology Department, Faculty of Pharmacy, Alexandria University, El-Khartoom Square, Azarita, Alexandria Egypt

**Keywords:** Uropathogenic *E. coli*, Quorum sensing, Repurposing, Carvacrol, Cinnamaldehyde, Eugenol, Egypt

## Abstract

**Background:**

Urinary tract infections represent one of the most frequent hospital and community-acquired infections with uropathogenic *Escherichia coli* (UPEC) being the main causative agent. The global increase in the emergence of multidrug-resistant (MDR) UPEC necessitates exploring novel approaches. Repurposing natural products as anti-quorum sensing (QS) agents to impede bacterial virulence is gaining momentum nowadays. Hence, this study investigates the anti-QS potentials of carvacrol, cinnamaldehyde, and eugenol against *E. coli* isolated from urine cultures of Egyptian patients.

**Results:**

Antibiotic susceptibility testing was performed for 67 *E. coli* isolates and 94% of the isolates showed MDR phenotype. The *usp* gene was detected using PCR and accordingly, 45% of the isolates were categorized as UPEC. Phytochemicals, at their sub-inhibitory concentrations, inhibited the swimming and twitching motilities of UPEC isolates, with eugenol showing the highest inhibitory effect. The agents hindered the biofilm-forming ability of the tested isolates, at two temperature sets, 37 and 30 °C, where eugenol succeeded in significantly inhibiting the biofilm formation by > 50% at both investigated temperatures, as compared with untreated controls. The phytochemicals were shown to downregulate the expression of the QS gene (*luxS*) and critical genes related to motility, asserting their anti-QS potential. Further, the combinatory activity of the phytoproducts with five antibiotics was assessed by checkerboard assay. The addition of the phytoproducts significantly reduced the minimum inhibitory concentrations of the antibiotics and generated several synergistic or partially synergistic combinations, some of which have not been previously explored.

**Conclusions:**

Overall, carvacrol, cinnamaldehyde, and eugenol could be repurposed as potential anti-QS agents, which preferentially reduce the QS-based communication and attenuate the cascades of gene expression, thus decreasing the production of virulence factors in UPEC, and eventually, subsiding their pathogenicity. Furthermore, the synergistic combinations of these agents with antibiotics might provide a new perspective to circumvent the side effects brought about by high antibiotic doses, thereby paving the way for overcoming antibiotic resistance.

**Supplementary Information:**

The online version contains supplementary material available at 10.1186/s12866-023-03055-w.

## Background

Urinary tract infections (UTIs) are considered among the most contracted bacterial illnesses worldwide throughout the community and hospital settings [1]. They are associated with high mortality and morbidity imposing massive economic and societal burdens [2]. Uropathogenic *Escherichia coli* (UPEC), the main etiological agent of UTIs, is responsible for more than 80% of UTIs globally [3]. Over the last decades, the problem has escalated with the emergence of a multidrug-resistant (MDR) phenotype among UPEC, featuring an alarming situation [4]. In Egypt, a comparable context is noticed. The easily attainable antibiotics without prescription and their extensive usage in clinical treatment, or as boosters in crop and livestock production, have triggered the emergence of elevated resistance rates among UPEC isolates. This classified Egypt as a country with the highest detected resistance levels compared to its neighboring counterparts in the Arab League [5, 6].

The exceptional ability of the UPEC strains to establish UTI is majorly endorsed by the production of a wide array of virulence factors. These include several adhesins known to increase the colonizing potential of UPEC, as well as an arsenal of weaponries such as toxins, proteases, and siderophores. The iron-chelating compounds secreted by a UPEC strain enable the uropathogen to overcome the restriction of iron availability and to survive inside the nutritionally deficient bladder environment [7]. Moreover, recurrent and difficult-to-treat UTIs are provoked by the UPEC’s capability to form biofilm, a sophisticated defense mechanism creating a sessile bacterial community of cells immersed in a matrix of extracellular polymeric substances creating dormant bacterial repository from which bacteria can re-emerge causing recurrence, thus posing a substantial challenge to physicians [8].

The production of virulence factors in *E. coli* is fostered and strictly regulated by a cell density-dependent bacterial signaling system. This phenomenon is collectively named quorum sensing (QS) [9, 10]. QS is initiated through the secretion of chemical signaling molecules known as autoinducers. When these autoinducers reach certain thresholds, a signal transduction cascades, and the expression of certain genes is triggered, resulting in a particular change in bacterial behavior. This change may involve antimicrobial resistance (AMR), bacterial bioluminescence, toxin secretion, formation of biofilm, or altered motility [11]. It has been reported that *E. coli* possesses five major QS systems namely LuxS/autoinducer-2 (AI-2), AI-3/epinephrine/norepinephrine, indole signaling, extracellular death factor, and SdiA QS systems. These QS circuits participate in regulating virulence genes mediating biofilm production, mobility, toxicity, and the production of curli [12].

For decades, the cornerstone strategy for the treatment of UTIs has relied upon the administration of conventional antibiotics such as fosfomycin, nitrofurantoin, sulfamethoxazole/trimethoprim, fluoroquinolones, in addition to aminoglycosides and tetracyclines [7, 13]. However, the rising resistance of UPEC isolates to these routinely used antibiotics is becoming a significant health issue that necessitates a prompt discovery of new alternatives [14]. One of the promising approaches for “disarming” the bacterial virulence is the anti-QS approach. Numerous efforts have been made to create quorum-sensing inhibitors (QSIs) from natural compounds [12, 15]. It has been demonstrated that grape seed extract acts as a natural QSI in *E. coli* via decreasing flagellar motility and Shiga toxin production, while extracts of broccoli, oregano, rosemary, turmeric, ginger, and basil inhibit the swarming motility of *E. coli* by impairing the AI-2 QS system [12, 16]. It seems that hijacking QS signaling using QSIs does not only halt bacterial resistance but also tends to disrupt the production of the associated virulence genes without affecting bacterial fitness [11]. Therefore, the anti-QS strategy can be regarded as an attractive alternative that does not exert the classical selective pressure and is able to tackle virulence, especially when used in synergistic combinations with conventional antibiotics [17]. In the current study, we attempted to investigate the ability of some essential phytoproducts, namely carvacrol (CaRV), cinnamaldehyde (CiNN), and eugenol (EG), to suppress the QS-mediated virulence factors such as biofilm formation and motility in UPEC isolated in Egypt. Subsequently, we present an in vitro assessment of the combined effect of these agents with selected antibiotics as a solution to combat the MDR phenotype widely encountered among UPEC isolates.

## Results

### Identification of the tested isolates and demographic profiles of study subjects

**Fig. 1:**
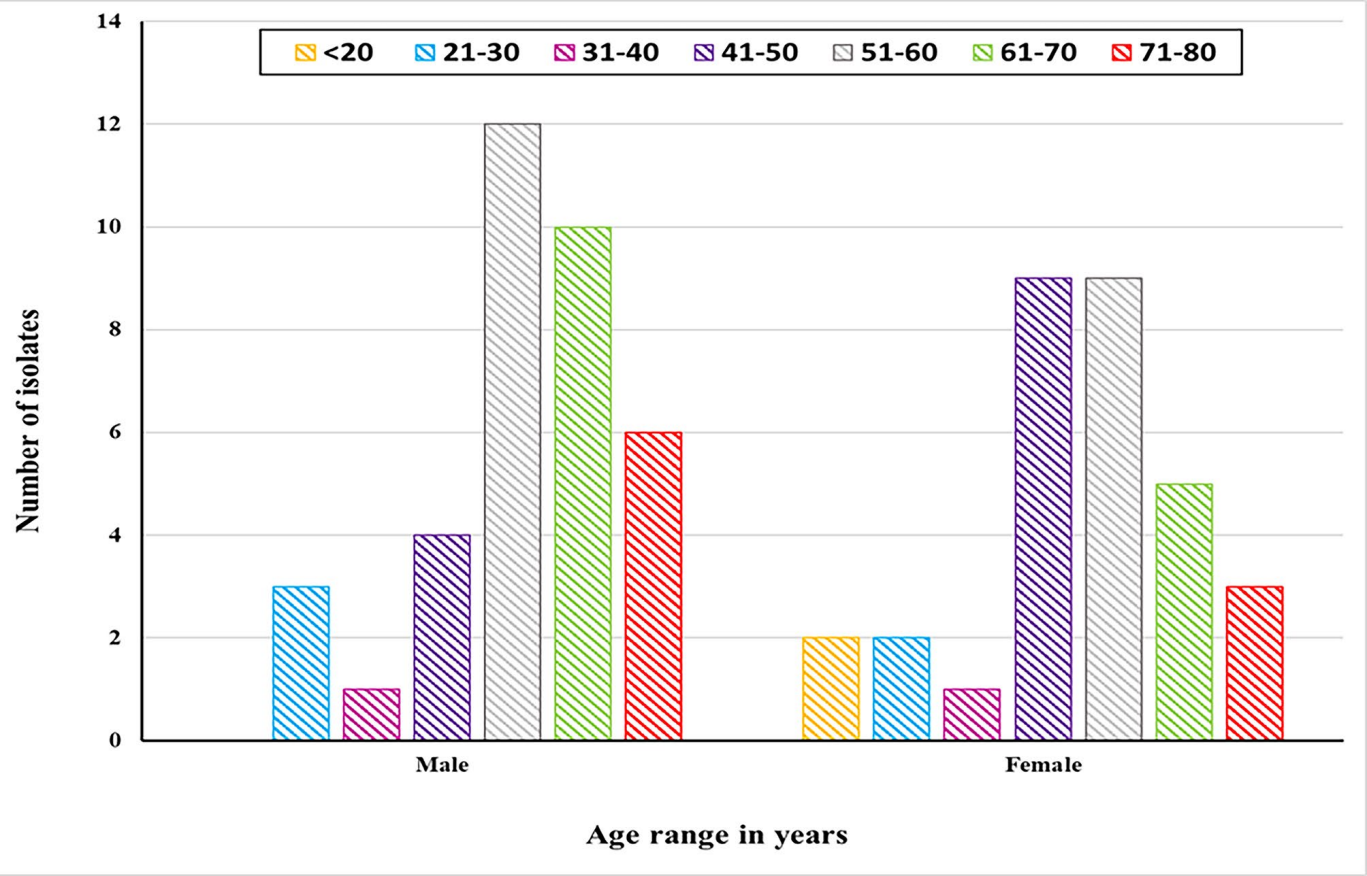
Distribution of *E. coli* specimens according to the age groups and gender of patients

The identity of the 67 isolates collected from the routine laboratory of Alexandria Main University Hospital (AMUH) was confirmed to be *E. coli* as indicated by the biochemical tests illustrated in Additional file 1. The demographic characteristics of 67 patients (36 males and 31 females) from whom the specimens were isolated showed that UTIs were more prevalent among males in the fifth decade with no incidence in the age group below 20, whereas females showed the highest prevalence in the fourth- and fifth-decade groups (Fig. 1).

### Assessment of antibiotic resistance and the effect of the phytochemicals on the growth of *E. coli* isolates

**Fig. 2:**
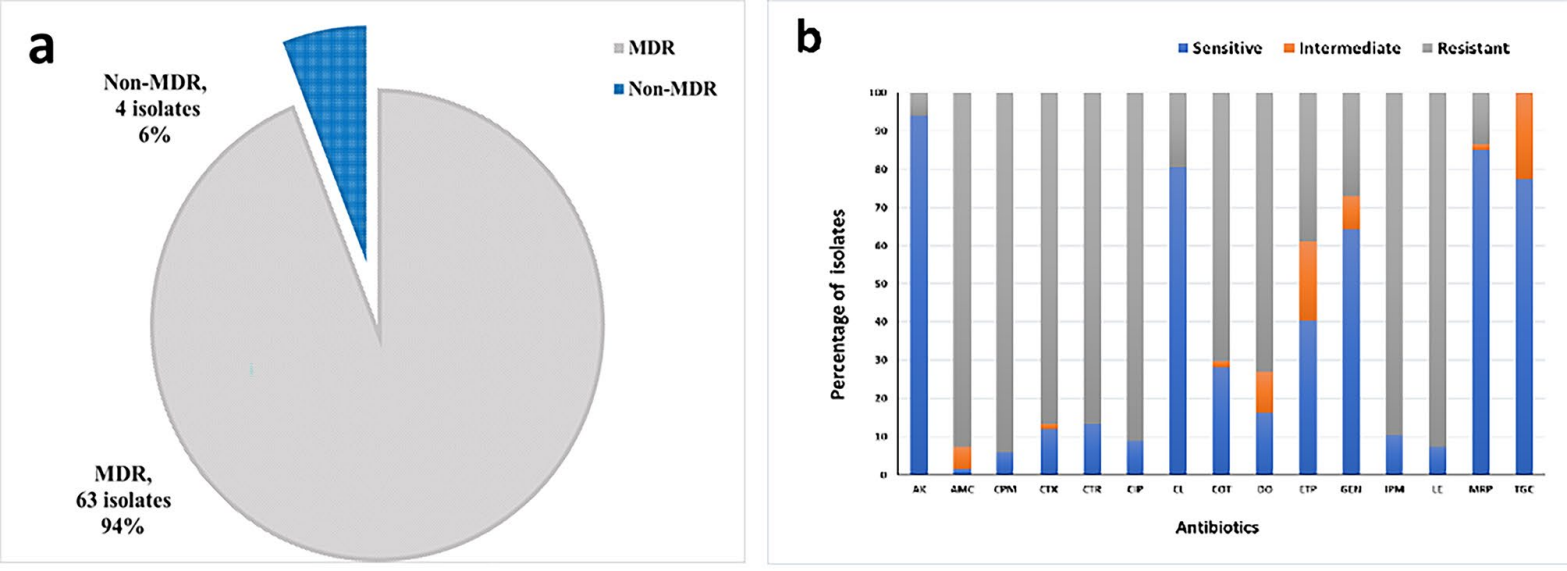
**(a)** Prevalence of multidrug-resistant (MDR) phenotype, **(b)** Antibiotic resistance profiles of 67 *E. coli* isolates. Antibiotics include AK: amikacin, AMC: amoxicillin/clavulanate, CPM: cefepime, CTX: cefotaxime, CTR: ceftriaxone, CIP: ciprofloxacin, CL: colistin, DO: doxycycline, ETP: ertapenem, GEN: gentamicin, IPM: imipenem, LE: levofloxacin, MRP: meropenem, TGC: tigecycline, and COT: sulfamethoxazole/trimethoprim

The susceptibility of 67 *E. coli* isolates to fifteen antibiotics, selected among the recommended ones for UTIs, was determined using the Kirby–Bauer disk diffusion method. The results showed that a very high percentage of these isolates, reaching 94%, displayed an MDR phenotype, being resistant to at least one agent in three or more chemical classes of antimicrobials (Fig. 2a). More than 70% of the isolates were resistant to amoxicillin/clavulanate, cefepime, cefotaxime, ceftriaxone, imipenem, doxycycline, sulfamethoxazole/trimethoprim, ciprofloxacin, and levofloxacin. The highest percentage of susceptibility was detected to amikacin (94%), followed by meropenem (85%), then colistin (80.5%). None of the isolates was resistant to tigecycline; nevertheless, 22.4% of the isolates were intermediately resistant to it (Fig. 2b). The different antibiotic resistance patterns obtained for the 67 isolates as well as their multiple antibiotic resistance (MAR) indices are represented in Additional file 2. A percentage of 94% of the isolates had a MAR index greater than 0.2 indicating that the organisms were isolated from a region with an upsurge in MDR pathogens. Among the phytochemicals, CiNN exhibited the highest activity level with a minimum inhibitory concentration (MIC) range of 130 to 260 µg/mL, followed by CaRV (MIC range: 250–1000 µg/mL), then EG which yielded an MIC range of 800 to 1600 µg/mL (Additional file 3).

### Antibiotic-antibiotic pairwise correlations

**Fig. 3:**
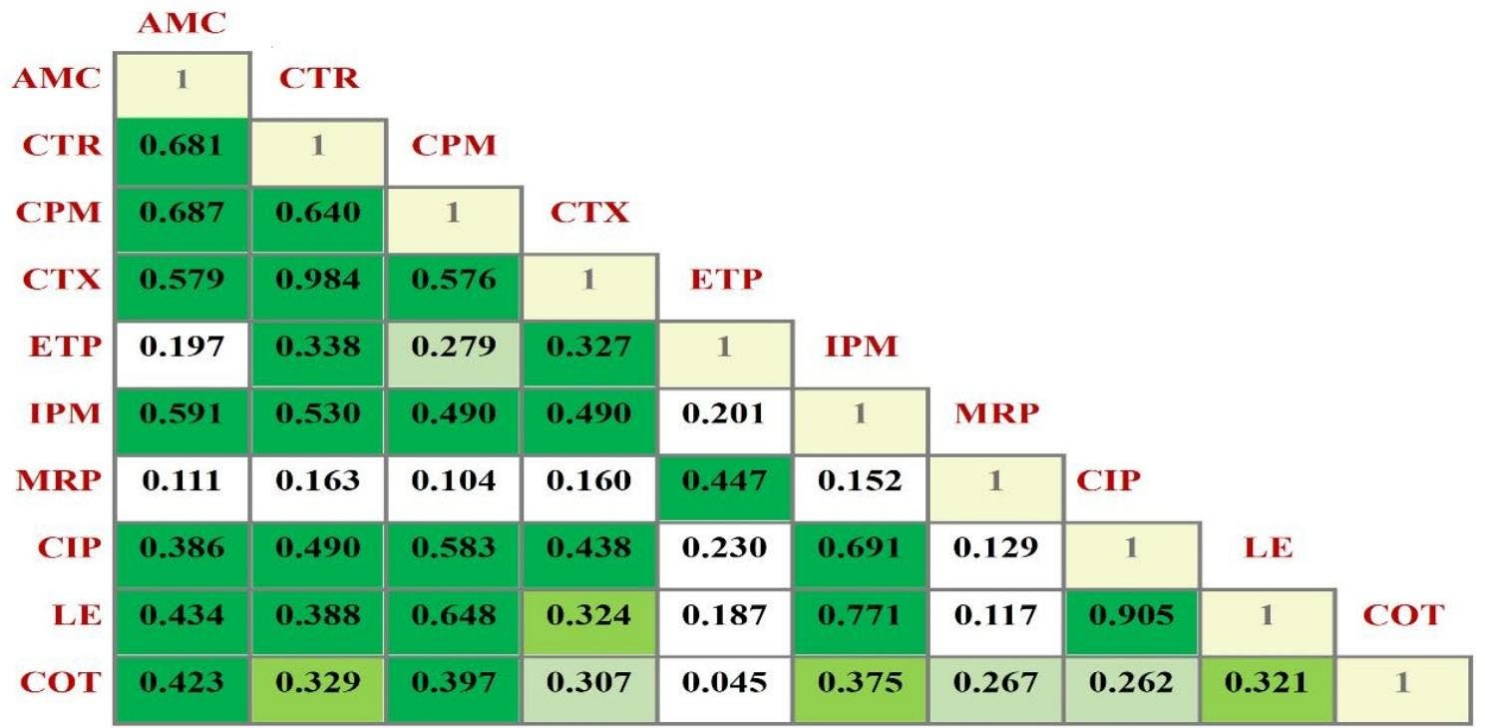
Correlation matrix showing Pearson’s correlation coefficients (r) calculated based on the resistance profile obtained from 67 *E. coli* isolates. The dark-colored green, moderately colored green, and light-colored green boxes denote correlations that are significant at *p* ≤ 0.001, *p* ≤ 0.01, and *p* ≤ 0.05, respectively. β-lactams include AMC (amoxicillin/clavulanate), CTR (ceftriaxone), CPM (cefepime), CTX (cefotaxime), ETP (ertapenem), IPM (imipenem), and MRP (meropenem). CIP (ciprofloxacin) and LE (levofloxacin) belong to fluoroquinolones, while COT (sulfamethoxazole/trimethoprim) belongs to the folate pathways antagonists’ class

Resistance to two or more antibiotics belonging to the same class is referred to as cross-resistance and is thought to be caused by a similar genetic mechanism. Conversely, associated resistance is defined as resistance to two or more antibiotics belonging to different classes and is deemed to be caused by unrelated mechanisms [18]. To explore the pairwise correlation between different antibiotics used in the current study, the resistance data of the 67 isolates was used to build a correlation matrix based on Pearson correlation coefficient (Fig. 3). The matrix implied that ceftriaxone–cefotaxime, cefepime–amoxicillin/clavulanate, and ceftriaxone–amoxicillin/clavulanate pairs were the most correlated and showed an extremely high significance at *p* ≤ 0.001. A strong correlation was observed as well among the tested fluoroquinolones (levofloxacin-ciprofloxacin) with Pearson correlation coefficient of 0.905 (*p* ≤ 0.001).

### Molecular detection of *usp* gene

**Fig. 4:**
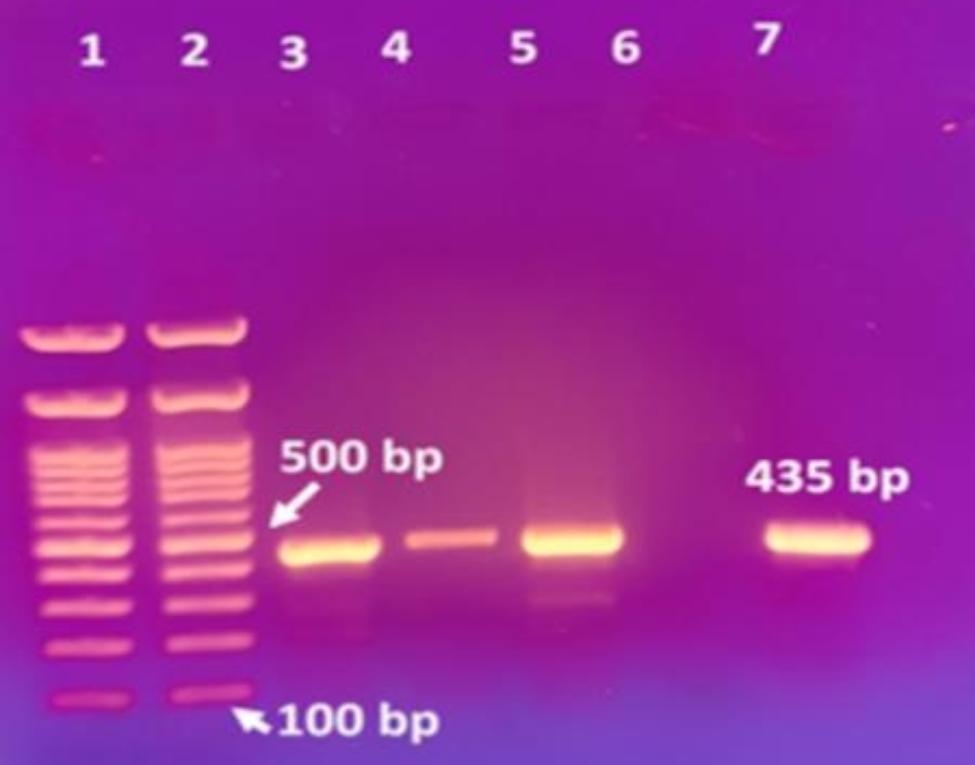
Representative agarose gel showing PCR amplification of *usp* gene in selected *E. coli* isolates. Lanes 1 and 2 correspond to a DNA molecular marker (100 bp). Lanes 3, 4, 5, and 7 show the amplicon (435 bp) corresponding to *usp* gene in E1, E2, E3, and E10 isolates, respectively. Lane 6 corresponds to the result of E5, a non-*usp-*harbouring isolate

PCR analysis identified an amplicon of 435 bp corresponding to the *usp* gene which codes for the uropathogenic specific protein in 30/67 of the tested *E. coli* isolates, thus classifying 45% of the isolates as UPEC. A representative agarose gel showing the PCR amplification of *usp* gene in some selected *E. coli* isolates is illustrated in Fig. 4.

### Quorum sensing inhibition assays

#### Inhibition of swimming and twitching motilities

**Fig. 5:**
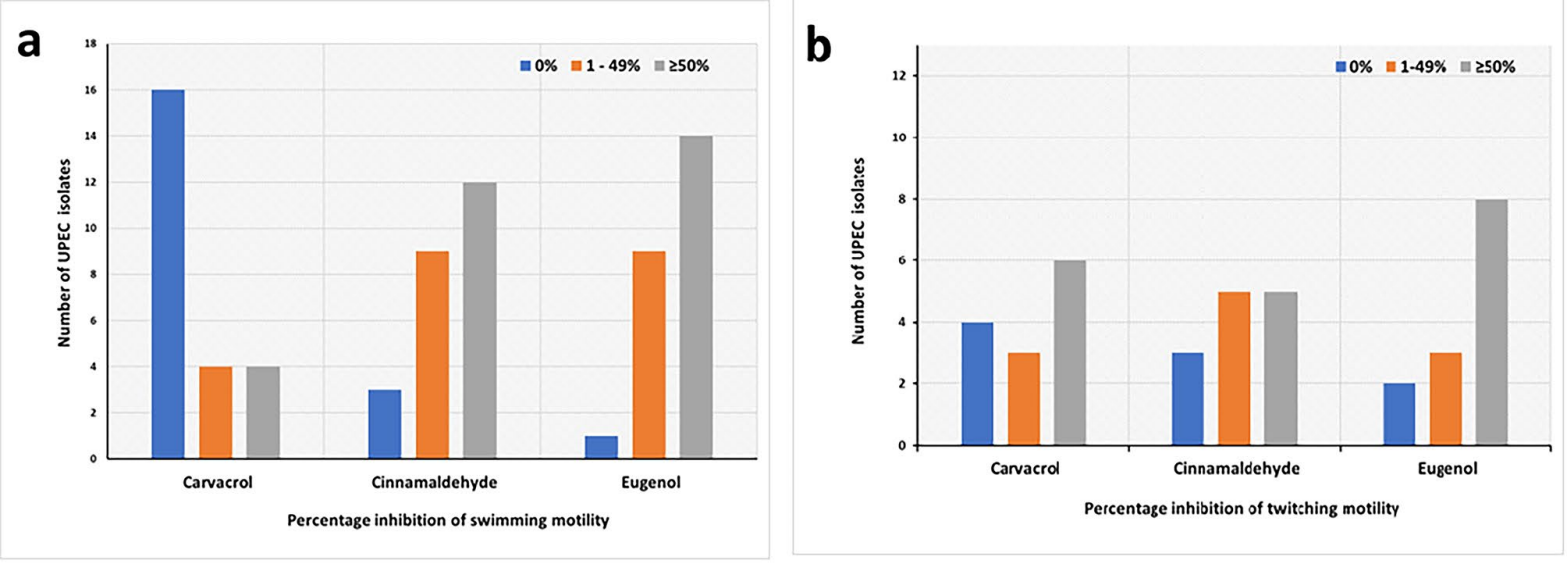
Impact of carvacrol, cinnamaldehyde, and eugenol at their sub-MICs on the quorum sensing-mediated **(a)** Swimming motility, and **(b)** Twitching motility of the tested UPEC isolates

**Fig. 6:**
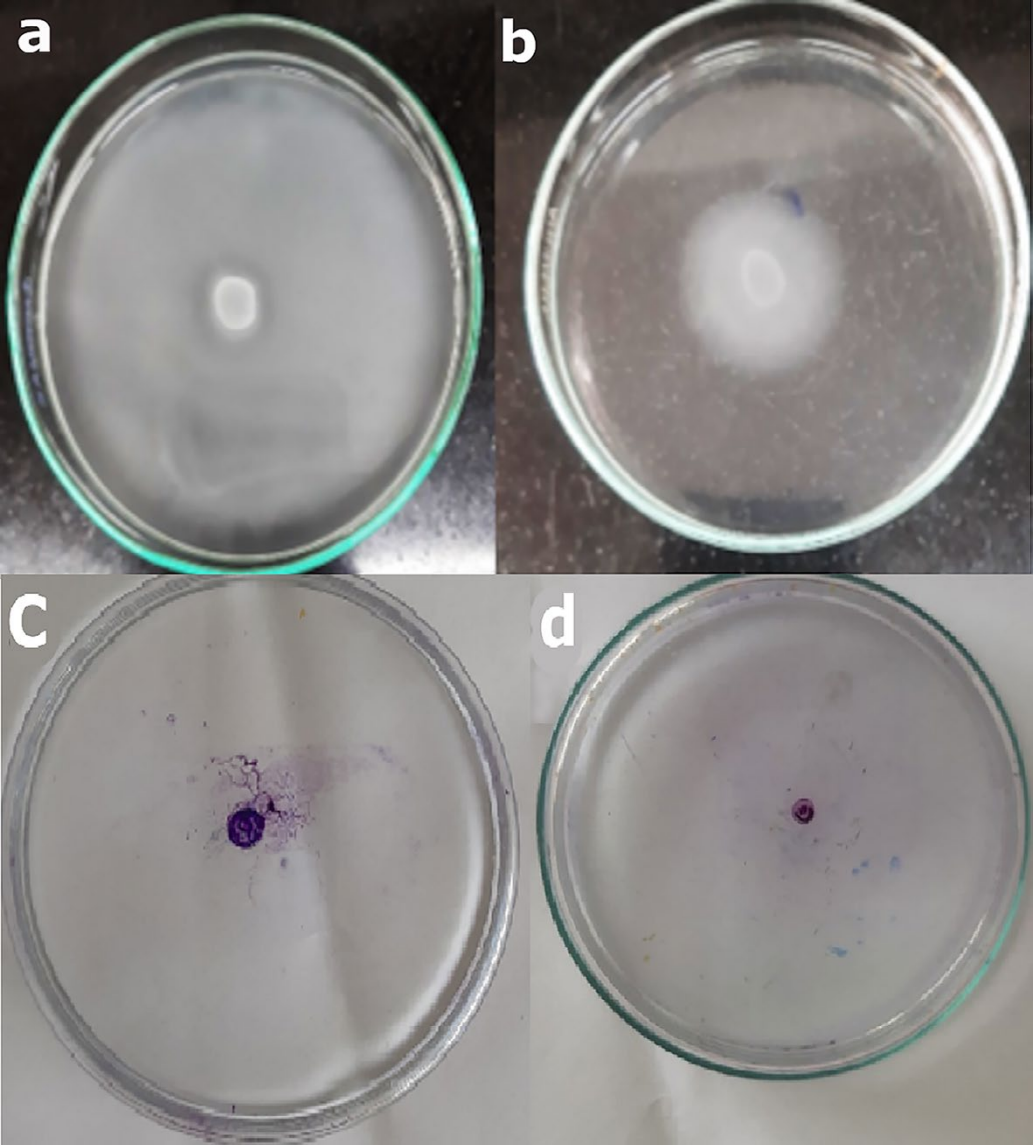
Inhibitive effect of cinnamaldehyde on the swimming motility of E51 isolate showing **(a)** Control without cinnamaldehyde and **(b)** Cinnamaldehyde at a concentration equivalent to 0.5XMIC; Inhibition of twitching motility of isolate E63 by eugenol showing **(c)** Control without eugenol and **(d)** Eugenol at a concentration corresponding to 0.5XMIC

The effect of 0.5XMIC of CaRV, CiNN, and EG on the swimming motility was investigated in 24/30 UPEC isolates that originally exhibited a halo zone diameter of swimming motility of ≥ 40 mm. The isolates were grouped into 3 subgroups, where group 1 included isolates that did not show any suppression by the tested agent, group 2 comprised isolates undergoing an inhibition range of 1–49%, while group 3 encompassed isolates having their swimming motility hindered in the presence of the agents by a value of ≥ 50%. Various inhibition patterns were observed, where EG and CiNN were able to suppress the swimming motility of 14/24 and 12/24 of UPEC isolates, respectively, by a value of ≥ 50%. On the other hand, CaRV showed the least inhibitory effect as it was unable to repress the swimming motility of 16/24 of UPEC isolates (Fig. 5a). To test the inhibitory effect of the phytochemicals on the twitching motility, intermediately motile isolates having twitching zone diameters of 5–20 mm were selected to build Fig. 5b. At concentrations corresponding to their 0.5XMIC, EG possessed the highest inhibitory effect followed by CaRV, then CiNN, where 8/13, 6/13, and 5/13 of UPEC isolates, respectively, had their twitching motility inhibited by a value of ≥ 50%. Representative illustrations showing the inhibition of swimming and twitching motilities by phytochemicals are presented in Fig. 6.

#### Impeding the biofilm formation

**Fig. 7:**
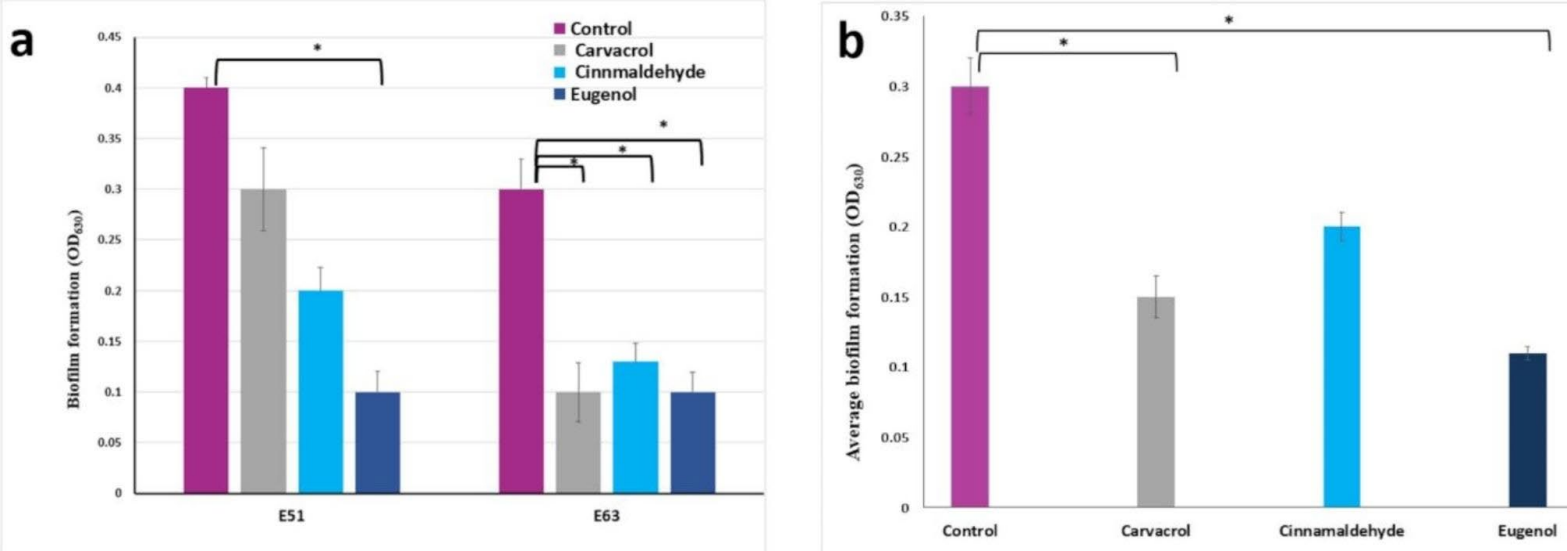
Anti-biofilm activities of carvacrol, cinnamaldehyde, and eugenol at their sub-MICs in: **(a)** E51 and E63 UPEC isolates experimented at 37 ºC, **(b)** 20 UPEC isolates investigated at 30 ℃. Data are shown as a mean of three independent experiments with error bars indicating SDs. The *p*-values indicate the significance at *p < 0.05 vs. non-treated samples

The inhibitory prospects of 0.5XMIC of CaRV, CiNN, and EG on the biofilm-forming ability were assessed at two temperature sets, 37 and 30 ºC, in isolates showing OD_630_ above 0.1 (2X ODc). At 37 ºC, EG exhibited the highest suppression level among the tested agents, where it hindered biofilm formation in E51 (initially a strong biofilm-forming isolate) by 75% and in E63 (a moderate biofilm-forming isolate) by 67%, both inhibition values being significant at *p* < 0.05 (Fig. 7a). For the assay at 30 ºC, 20 UPEC isolates exhibiting strong to moderate biofilm-forming capacity were selected and average biofilm formation was calculated for the control or in the presence of the phytochemicals (Fig. 7b). Eugenol succeeded in significantly inhibiting the biofilm formation by 67% (*p* < 0.05), followed by CaRV which significantly decreased the biofilm-forming ability by 50%, then CiNN showing a percentage inhibition of 33% as compared with untreated controls (Fig. 7b).

### Differential expression levels of genes

**Fig. 8:**
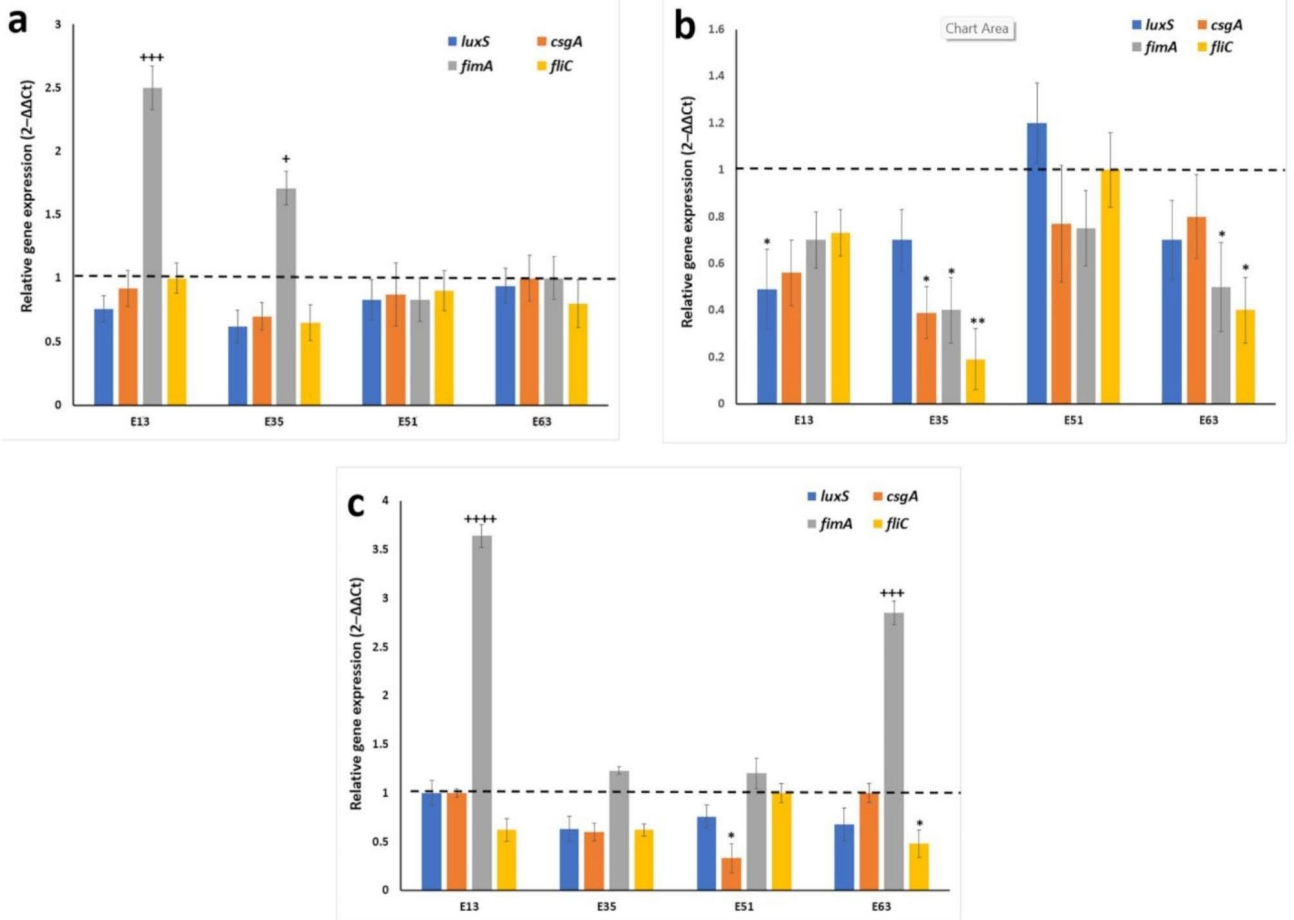
Relative expression of QS gene (*luxS*) and QS-regulated virulence genes (*csgA*, *fimA*, and *fliC*) associated with the production of virulence factors in UPEC, following treatment with sub-MICs of **(a)** Carvacrol, **(b)** Cinnamaldehyde, and **(c)** Eugenol. Gene expression was normalized with *gapA* and represented relative to the expression of genes in the untreated control. The error bars indicate SDs. The *p*-values indicate the significance of fold decrease at *p < 0.05 and fold increase at ^+++^*p* < 0.001 and ^++++^*p* < 0.0001

qRT-PCR analysis was carried out to assess the ability of CaRV, CiNN, and EG, at their sub-MIC range, to suppress the expression levels of the QS gene (*luxS*) and the QS-regulated virulence genes. These included the genes coding curli fimbriae (*csgA*), a crucial component for biofilm formation, a fimbrial adhesin gene, *fimA*, and *fliC*, a flagellar gene essential for the motility of UPEC. Four isolates were selected for the transcriptional analysis: E13, E35, E51, and E63 isolates. The selection was based on the isolates evident display of phenotypic expression of biofilm formation and motility. To allow the maximum expression of their virulence factors, E51 and E63 were grown at 37 ºC, while E13 and E35 were subcultured at 30 ºC in the presence and absence of the agents. The phytochemicals commonly altered the expression levels of the tested genes, yet in a variable manner. Carvacrol succeeded in downregulating the major QS gene, *luxS*, in 100% of the tested isolates with a fold reduction ranging from 1.06 (in E63 isolate) to 1.62 (in E35 isolate). *csgA* gene was repressed by CaRV in 75% of the isolates and the repression ranged from 1.1- (in E13 isolate) to 1.4-fold (in E35 isolate). *fliC* gene was downregulated in 75% of the isolates with the highest fold reduction reaching 1.5-fold in E35 isolate and the lowest fold reduction reaching 1.1 in E51 isolate. Carvacrol reduced the expression level of *fimA* in a single isolate, E51, by 1.2-fold. (Fig. 8a). CiNN repressed the expression levels of *csgA* and *fimA* in 100% of the isolates with a fold reduction ranging from 1.3 to 2.6. It reduced the levels of *luxS* and *fliC* in 75% of the isolates with a fold reduction ranging from 5.3 to 1.3 (Fig. 8b). The levels of transcripts of *luxS* and *fliC* were reduced by an average of 1.46- and 1.76-fold, respectively, in 75% of the isolates by EG and those of *csgA* were downregulated in 50% of the tested isolates by a fold reduction ranging from 1.67 (in E35 isolate) to 3 (in E35 isolate). An unexpected significant upregulation of *fimA* gene was noticed upon treatment with CaRV or EG (Fig. 8a and c, respectively).

### Assessment of the combined activity of phytochemicals and antibiotics by checkerboard assay

The combined effect of CaRV, CiNN, and EG with ciprofloxacin, ceftriaxone, colistin, doxycycline, and tigecycline was investigated against E18, E35, and E63 isolates. These isolates were chosen based on their MDR phenotype, being resistant to 9 antibiotics belonging to 4 different classes. The results in Table 1 are expressed in terms of fractional inhibitory concentration (FIC) index and combinations displaying synergy or partial synergy are referred to as successful ones. The addition of the phytoproducts significantly reduced the MIC of the antibiotic as demonstrated by the fold reduction in the MICs of the used antimicrobials (Additional file 4). Carvacrol generated successful combinations with ceftriaxone, colistin, or doxycycline in 100% of the tested isolates. When combined with tigecycline, CiNN showed partial synergy in 66.6% of the isolates, while EG revealed successful combinations with ciprofloxacin, ceftriaxone, or tigecycline in 100% of the isolates included in the assay.

**Table 1:** FIC index for the combined activity of the phytochemicals with antibiotics against UPEC isolates

	FIC index (Interpretation)														
	Carvacrol +					Cinnamaldehyde +					Eugenol +				
	CIP	CTR	CL	DO	TGC	CIP	CTR	CL	DO	TGC	CIP	CTR	CL	DO	TGC
E18	1 (Add)	0.27 (Syn)	0.53 (PSyn)	0.56 (PSyn)	1 (Add)	1.25 (Ind)	0.75 (PSyn)	1.25 (Ind)	0.56 (PSyn)	0.75 (PSyn)	0.625 (PSyn)	0.75 (PSyn)	1.25 (Ind)	1.25 (Ind)	0.53 (PSyn)
E35	1 (Add)	0.75 (PSyn)	0.5 (Syn)	0.625 (PSyn)	1 (Add)	1 (Add)	1 (Add)	0.75 (PSyn)	1.25 (Ind)	1.25 (Ind)	0.75 (PSyn)	0.75 (PSyn)	1 (Add)	1 (Add)	0.5 (Syn)
E63	0.75 (PSyn)	0.625 (PSyn)	0.75 (PSyn)	0.75 (PSyn)	1.25 (Ind)	1.12 (Ind)	1 (Add)	1 (Add)	1 (Add)	0.625 (PSyn)	0.625 (PSyn)	0.5 (Syn)	0.75 (PSyn)	0.56 (PSyn)	0.75 (PSyn)

CIP: ciprofloxacin, CTR: ceftriaxone, CL: colistin, DO: doxycycline, TGC: tigecycline. Ind, Add, PSyn, and Syn indicate indifference, additive effect, partial synergy, and synergy, respectively

## Discussion

Globally, 150 million UTI cases are reported each year, thus increasing the medical care expenses [19]. Although other bacteria may cause UTIs, UPEC is by far the most predominant pathogen infecting the urinary tract in the United States, Europe, and Latin America [20, 21]. In the current study, 67 *E. coli* isolates were collected from the urine cultures of patients with UTIs admitted to AMUH from July to October 2018. Demographically, a higher incidence of UTIs in male patients (54%) as compared to their female counterparts (46%) was recorded in this investigation. Although females are known to be at a higher risk group for developing UTIs owing to the anatomical structure of their urinary system [22], other observers found that these infections were more common in males [23]. This could be explained by the higher age group of the patients, exceeding 40 yrs, from whom the samples were collected in this study. In older age, males are at higher risk of catheterization, prostate gland enlargement, and reduced bacteriostatic secretions of the prostate, all representing factors elevating the incidence of UTIs [24].

The treatment of UTIs is usually achieved by a short-term antibiotic regimen, but bacterial resistance to antimicrobials is escalating. A percentage of 94% of the isolates in this study exhibited an MDR phenotype. This elevated incidence rate is in accordance with the rates reported in other parts of Egypt, where MDR *E. coli* was detected among 96%, 91%, and 95% of the tested isolates in Minia, Dakahlia and Giza, and Cairo, respectively [25–27]. MAR indexing method, a useful technique for the evaluation of risk assessment, provides an estimate of the number of bacteria that exhibit antibiotic resistance in the risk area throughout any standard susceptibility testing [28]. A MAR index of > 0.2, a value identical to the one reported by Masoud et al. in 2022 in Egypt [25], was detected for 94% of the isolates in our study. This high MAR index implies the prevalence of persistent pathogens that are resistant to conventional antibiotics at the collection area. Self-medication with the lack of underlying adequate tests, improper antibiotic use, and blind diagnosis are condemned for this extremely high value [29]. More than 85% of the isolates in our collection showed resistance to cephalosporins. These high levels of resistance, endorsed by the World Health Organization report in 2014 are thought to be attributed to their low cost, simple access, and frequent prescription in Egyptian healthcare establishments. While 89.5% and 38.8% of the isolates were resistant to imipenem and ertapenem, respectively, meropenem remained resilient against most of the isolates, with a percentage of susceptibility of 85%. Meropenem is not frequently prescribed in outpatient clinics and is regarded as a last-resort antibiotic in Egyptian hospitals [30]. However, the emergence of carbapenem-resistant *E. coli* strains is noticed globally necessitating the prudent use of this antibiotic [31]. In addition to meropenem, our results highlight the effectiveness of aminoglycosides, tigecycline, and colistin as treatment options for the eradication of resistant urinary *E. coli* isolates.

Analyzing the pairwise correlations, highly significant correlations (*p* ≤ 0.001) were detected among β-lactams pairs and the fluoroquinolones pair (ciprofloxacin-levofloxacin) referring to the phenomenon of cross-resistance. A rational justification by Mohamed et al. [32]. indicated that isolates tend to display similar resistance to antibiotics with related chemical structures. Whereas the correlations detected between the β-lactams-fluoroquinolones and β-lactams-co-trimoxazole pairs refer to associated resistance and are explained by the fact that the implicated resistance genes are co-existing on plasmids that can be easily transferred among bacterial strains [30, 33].

The pathogenicity of a UPEC strain fundamentally depends on the production of virulence factors including adhesins, toxins, siderophores, destructive enzymes, and persistent biofilms that are regulated by its QS mechanism [34]. The last decade has witnessed a revolutionary increase in AMR worldwide, urging the need to discover novel non-antibiotic treatment approaches [35]. In this context, tackling the bacterial QS circuits to constrain virulence has gained attention among researchers, paving the way for novel anti-virulence strategies [12]. The anti-QS approach was pioneered in 2006 by Niu et al. [36]., where the authors demonstrated the CiNN’s ability to impede QS in *Vibrio harveyi* via modulating the expression of QS genes. Since then, reports emphasizing the anti-QS potentials of different natural phytoproducts and their capacity to attenuate virulence in different microorganisms have surfaced [37–40]. However, scarce data are available regarding the effect of CaRV, CiNN, and EG on the virulence of MDR UPEC isolates and their mechanism of QS inhibition in these isolates. Hence, the current study focused on evaluating the anti-virulent potential and anti-QS activity of these three phytochemicals against UPEC isolates. We initiated the study by determining the MICs of CaRV, CiNN, and EG, followed by the selection of the sub-MIC ranges to allow the subsequent study of their anti-QS efficacies. Previous observations indicated that subinhibitory concentrations of CaRV, CiNN, and EG inhibited the motility and biofilm-forming ability, traits known to be regulated by QS in *E. coli*, in a dose-dependent manner [41–43]. These observations are consistent with our results, where higher concentrations demonstrated antimicrobial activities of the agents, while lower ones showed anti-QS abilities. Further, we examined the anti-motility impact of the agents against UPEC. Apart from playing a fundamental role in the colonization of the urinary tract and biofilm formation, motility enables UPEC to avoid the wash-out effect of the urine flow, evade the host defense mechanisms, and compete with other pathogens in polymicrobial niches where the advantage is given to motile pathogen [35]. In accordance with previous studies [44, 45], the tested phytochemicals at their sub-MICs succeeded in decreasing the swimming motility of UPEC by a value of ≥ 50%.

Twitching motility is considered one of the prerequisites for biofilm formation [46] and while being studied intensively in other pathogens such as *Pseudomonas aeruginosa*, *P. fluorescens*, and *Erwinia carotovora* [35, 47], to the best of our knowledge, this is the first investigation shedding light on the phenotypic inhibitory effect of the three phytochemicals on the twitching motility in UPEC. The phytoproducts reduced the twitching motility of the tested isolates by ≥ 50%, thus facilitating the hindrance of the succeeding step, biofilm formation.

The biofilm-forming ability of the UPEC isolates was observed to be enhanced at 30℃ when compared to their ability at 37℃. An increased biofilm formation at 30℃ was reported as well by Uhlich et al. [48]. in some *E. coli* pathotypes. This could be explained by the fact that curli synthesis, an important attribute for biofilm formation, is temperature-dependent and that 30℃ is considered to be the optimum temperature for its expression as reported by Leech et al. [49]. In their study, Leech et al. [49]. demonstrated that the highest expression of *csg* genes occurred at 30 °C, followed by 28 °C, then at 37 °C. Additionally, other fimbriae that contribute to biofilm formation, such as F9 fimbriae, are known to be optimally expressed at 28℃ [50]. The hazard of biofilm formation by UPEC isolates tends to augment with prolonged catheterization, during which, antibiotic treatment may worsen the situation by imposing selective pressure on the bacteria and leading to the acquisition of more resistance. Since QS circuits adopted by *E. coli* dominate the transition between the different phases of biofilm formation, an opportunity is created for an intervention using the anti-QS approach [51]. The abrogation of biofilm formation was tested in our study using two temperature sets, 30℃ and 37℃, a parameter not previously explored. The phytoconstituents were shown to hinder the biofilm formation in UPEC isolates at both temperatures demonstrating variable degrees of inhibition, with EG displaying the highest effect. This anti-biofilm effect of the agents at 37℃ is consolidated in the literature by several studies [42, 43, 52, 53].

Motivated by the anti-QS phenotypic results, we pursued to explore the genetic basis of this anti-QS effect, where the differential expression of the virulence-associated genes (*fimA, csgA*, and *fliC*) and the QS gene (*luxS*) were investigated under test and control conditions using qRT-PCR. The phytoproducts caused downregulation of *luxS* gene in all tested isolates implying that inhibition of the AI-2 system through *luxS* pathway is a suggested anti-QS mechanism exerted by these compounds. However, CiNN increased the expression level of *luxS* in a single isolate. This could be explained by the fact that downregulation of other genes engaged in the AI-2 QS system such as *pfs*, a gene that was not investigated in this study, could have occurred in this particular case [54]. The agents were able, as well, to repress the transcription of the curli fimbriae gene, *csgA*, a crucial component for biofilm formation, and *fliC*, a flagellar gene essential for the motility of UPEC. Our results were in accordance with previous reports investigating the expression levels of the abovementioned genes in UPEC or other *E. coli* pathotypes [41, 52, 55, 56]. Surprisingly, extremely significant upregulation of the fimbrial adhesin gene, *fimA*, was noticed in some isolates upon treatment with EG or CaRV. This result suggests that bacteria, when encountering unfavorable conditions in the environment, tend to selectively upregulate the expression of a certain gene, which is in this case the fimbrial adhesin gene, *fimA*, to counteract the suppressing effect exerted on another related gene.

Based on the results of the checkerboard assay, the combinatory effect of CaRV, CiNN, and EG with 5 antibiotics showed partial synergistic interactions in 53%, 33%, and 60% of the generated combinations, respectively. Synergistic combinations were detected, as well, when combining CaRV with ceftriaxone or colistin, and EG with tigecycline or ceftriaxone. Few data are available in the literature regarding the results of these combinations especially in UPEC isolates. Ibrahim and Al Meani [57] reported that combining clove oil, whose major component is EG, enhanced the effect of cefepime, ceftazidime, and ceftriaxone, whereas Vázquez-Ucha et al. [58] detected synergy between thyme oil, the major constituent of which is CaRV, and colistin in *Acinetobacter baumannii* and *Klebsiella pneumoniae*. In accordance with our results, a study conducted by Özel et al., in 2022, reported a synergistic interaction of EG with tigecycline in *E. coli*. The mode of action of CaRV and EG is thought to be due to their lipophilic nature which enables them to accumulate and disintegrate the lipids in the bacterial membrane causing its disruption, energy depletion, and leakage. Whereas CiNN binds to bacterial proteins preventing the action of important bacterial enzymes. Combining these natural compounds with antibiotics constrains bacterial adaptation and restores antibiotic sensitivity by allowing the penetration of the latter [59]. Another reason for the synergetic effect is accounted to the ability of EG and CaRV to disrupt the bacterial cytoplasmic membrane, permeabilize the cell wall, enhance the expression of porins, and downregulate the efflux pumps, thus facilitating the uptake of the antibiotics [60, 61]. Despite being terpenic components of essential oils, the effect of CaRV, CiNN, and EG vary due to the difference in their chemical structure, where the phenolic nature of CaRV and EG tend to exhibit a greater bactericidal effect when compared to aldehydes [62]. Consistent with our findings, CiNN displayed successful combinations with a lower number of antibiotics. The lack of the potentiating effect noted in some interactions can be ascribed to the presence of multiple genes of resistance with variable expressions [59]. The generated synergistic or partially synergistic combinations, some of which have not been previously explored, could provide a new perspective to overcome the side effects brought about by high antibiotic doses and minimize the problem of AMR.

## Conclusion

In conclusion, this study sheds light on the anti-QS potential of the phytoconstituents, CaRV, CiNN, and EG. Their combined use with conventional antibiotics succeeded in circumventing antibiotic resistance of MDR UPEC isolates paving the way to novel treatment options for the management of UTIs. Instead of exerting direct selective pressure on the growth of UPEC isolates, these phytochemicals could preferentially reduce the QS-based communication and attenuate the cascades of gene expression decreasing the production of virulence factors, and eventually, subsiding their pathogenicity. We understand and acknowledge the limitations of this study. The combined phytochemicals/antibiotic effect was investigated on a small sample size limiting the statistical significance of synergistic results. Further in vivo validation could have enabled the elucidation of the exact mechanism behind the anti-QS activity of these phytochemicals and their anti-virulent properties.

## Methods

### Collection and identification of *E. coli* clinical isolates

Sixty-seven *E. coli* clinical isolates were collected through the routine laboratory facility of AMUH from the urine cultures of patients admitted to the hospital with UTIs throughout the time duration of July to October 2018. The isolates were preserved as frozen stocks at -20 °C in Luria-Bertani broth (LB, HiMedia, Mumbai, India) containing 20% glycerol and were archived at − 80 °C. Before use, a fresh culture of each of the collected isolates was obtained by cultivation on MacConkey’s and eosin methylene blue agar (Oxoid, Hampshire, UK). Following incubation at 37 °C for 24 h, the isolated colonies were identified by Gram staining and then subjected to standard biochemical tests including triple-sugar iron, indole production, methyl red, Voges Proskauer, citrate utilization, catalase, and urease tests.

### Antimicrobial susceptibility testing

The susceptibility of the 67 *E. coli* isolates to 15 antimicrobials was determined by Kirby-Bauer disk diffusion method using cation-adjusted Mueller–Hinton (CAMH, HiMedia, Mumbai, India) agar. The following antibiotic disks were purchased from HiMedia Laboratories (Mumbai, India) and used for the test: amoxicillin/clavulanate, cefotaxime, ceftriaxone, cefepime, imipenem, meropenem, ertapenem, amikacin, gentamicin, doxycycline, tigecycline, ciprofloxacin, levofloxacin, sulfamethoxazole/trimethoprim, and colistin. The test was carried out according to the Clinical Laboratory Standards Institute (CLSI, 2021) guidelines using Mueller–Hinton agar (HiMedia Laboratories, Mumbai, India) and the results were interpreted in accordance with the breakpoints indicated in CLSI, except for tigecycline and colistin, where EUCAST (2018) and disk manufacturer’s guidelines were applied, respectively, to interpret the results. MAR index, a ratio between the number of antibiotics that an isolate is resistant to and the total number of antibiotics the isolate is exposed to, was calculated for the 67 tested *E. coli* isolates according to the method described by Masoud et al. [63]. Broth microdilution technique was performed to determine the MICs of the phytochemicals, CaRV (Sigma Aldrich, UK), CiNN (LOBA Chemie, Mumbai, India), and EG (LOBA Chemie, Mumbai, India). Prior to their use, the agents were sterilized using 0.45 μm bacterial filters (Filter-bio-CO., China) and were solubilized in 7.5% dimethyl sulfoxide (DMSO, Fischer Scientific, USA), a concentration which allowed the growth of the isolates as confirmed by the determination of DMSO MIC against all the tested isolates. Serial dilution of CiNN and EG was performed using 7.5% DMSO, while the stock solution of CaRV in 7.5% DMSO was serially diluted in sterile distilled water. The MIC values of the phytochemicals were determined by measuring the absorbance at OD_600_ after 24 h of incubation using an ELISA microtiter plate reader (BioTek 800 TS, USA) [64].

### Molecular detection of *usp* gene using PCR

The *usp* gene, coding for the uropathogenic specific protein, is regarded as a pivotal determinant in classifying *E. coli* strains as UPEC [65]. Therefore, the detection of the gene coding for Usp among the collected isolates was performed using PCR as previously discussed [66]. The following primers, obtained from Bio Basic Inc., Canada were used: *usp*-forward (5’-ACATTCACGGCAAGCCTCAG-3’) and *usp*-reverse (5’-AGCGAGTTCCTGGTGAAAGC-3’). The obtained PCR products were separated by gel electrophoresis in the presence of a 100-bp DNA ladder (GeneDireX RTU, Taiwan, yielding 11 fragments: 100–1500 bp). The bands were visualized on a 254 nm UV transilluminator (Entela UVP Upland CA 91,786, USA).

### Suppression of quorum sensing-regulated virulence factors by phytochemicals

The ability of CaRV, CiNN, and EG to inhibit the QS-mediated swimming and twitching motilities, as well as the biofilm-forming ability, was investigated in the *usp*-positive *E. coli* isolates.

### Swimming and twitching motilities assay

Swimming motility was assayed using 0.3% agar plates supplemented with 1% tryptone (Oxoid, Hampshire, UK) and 0.25% NaCl while twitching motility was experimented using LB broth containing 1% agar (B&V Srl, Italy), as previously described [43, 67]. CaRV, CiNN, EG, or DMSO (control) were added to the motility agar plates. The phytochemicals were incorporated at a subinhibitory concentration equivalent to 0.5XMIC of the agent calculated based on the MIC values for each isolate. For the swimming assay, aliquots of 1 µL of UPEC isolates grown to an OD_600_ of 1.0 were placed at the center of motility plates with or without the tested agents using sterilized micropipette tips. Diameters of swimming halos were measured following incubation at 37 °C for 24 h. In the twitching assay, a colony of each of the tested isolates was deeply stabbed using a sterile toothpick into the prepared plates with or without the phytochemicals in a way to touch the agar–dish interface. After incubation at 37 °C for 48 h, agar was removed and the adhered cells on the dish were stained with 1% (w/v) crystal violet solution. The violet-stained diameters were then accurately measured. *E. coli* ATCC 8739 and PAO1 were included in the tests as quality control strains.

### Crystal violet biofilm formation assay at 30 and 37 °C

The UTIs acquired in hospitals are mostly caused by using urinary catheters. Since the temperature may vary along the length of the catheters, where the collection bag is held at room temperature, the tubing is outside the urinary tract and closer to the skin, while the tip and initial tubing, being inserted in the bladder and the urethra, are subjected to body temperature, 37 °C. This variation in temperatures triggers the concern that UPEC may form biofilm at any part along the catheter. Therefore, the ability of the isolates to form biofilm at 37 ºC and 30 ºC was assessed using the microtiter plate assay as previously described with slight modifications [68]. Briefly, an inoculum of 200 µL of the overnight culture of each of the UPEC strains in nutrient broth (NB, Oxoid, Hampshire, UK) when testing at 37 ºC, or in LB broth when testing at 30 ºC, at an OD_600_ of 0.05 was introduced into a flat-bottomed 96-well microtiter plate (Sigma-Aldrich, USA) and incubated at 37 or 30 ºC for 48 h. To quantify the formed biofilms, they were stained for 10 min with crystal violet 0.2% (w/v), rinsed three times with distilled water, and extracted with 30% glacial acetic acid (or 95% ethanol in the assay at 30 ºC). Absorbances were measured at 630 nm, and results were expressed as the averages of three replicate wells. Subsequently, isolates that recorded an OD_630_ above 0.1 (2X ODc) indicating moderate to strong biofilm formation at both temperatures under test were investigated in the presence of the phytoconstituents at concentrations corresponding to 0.5XMIC, where a volume of 100 µL of an overnight culture of each of the UPEC isolates was added to 100 µL of the suitable culture media and incubated for 48 h. The procedure was then followed as mentioned above.

### Quantitative real-time PCR assay

For transcriptomic analysis, qRT-PCR was used to investigate the effect of CaRV, CiNN, and EG on the expression levels of the QS gene (*luxS*) and the virulence genes known to be modulated by QS (*csgA*, *fimA*, and *fliC*). Cultures of selected isolates were grown in LB broth for 48 h in the presence and absence of the agents at concentrations equivalent to 0.5XMIC. Bacterial pellets were harvested by centrifugation at 6000 rpm for 5 min at 4 ºC. Total RNA was extracted from treated and untreated cells using Biozol reagent™ (Bioflux, China) as per the manufacturer’s protocol and its concentration was adjusted by nanodrop (Thermo Fisher, USA). cDNA was synthesized using TOPscript™ RT DryMix (Enzynomics, South Korea) and then was quantified by qPCR using TOPreal™ SYBR® Green Premix (Enzynomics, South Korea) in Rotor-Gene Q Real-Time PCR system (Qiagen, Germany) with the primer pairs described in Online Resource Additional file 5. Each reaction mixture contained 1 µL of cDNA, 10 µL of SYBR® Green Premix, 1 µL of 10 µM of each primer, and 7 µL of nuclease-free water. To normalize the expression levels of the target genes, *gapA* was used as internal control and the relative gene expression was calculated according to Pfaffl method (2^−∆∆Ct^) [45]. The experiment was performed in triplicates and the results were denoted in terms of fold change.

### Checkerboard assays

The combined activity of CaRV, CiNN, and EG with ceftriaxone, ciprofloxacin, colistin, doxycycline, and tigecycline was analyzed by checkerboard assays against 3 UPEC isolates in 96-well microtiter plates. A volume of 50 µL of two-fold serial dilutions of the tested agents in 30% DMSO was introduced to each well of each row, whereas serial dilutions of the antibiotics were added to each well of each column. An overnight bacterial culture matching the turbidity of 0.5 McFarland was 100-fold diluted in double strength CAMH broth and a volume of 100 µL was inoculated into the checkerboard plates which were then incubated at 37 ºC for 24 h [69]. The FIC index was calculated to investigate the combined effect of the agents and antibiotics using the following equation: FIC of agent = MIC in combination/MIC alone, FIC index = FIC of antibiotic + FIC of phytochemical. For interpretation, an FIC index ≤ 0.5, denoted synergy; 0.5 < FIC index ≤ 0.75, indicated partial synergy; 0.76 < FIC index ≤ 1, designated additive effect; 1 < FIC index ≤ 4, showed an indifferent effect; while an FIC index > 4, referred to antagonism [70].

### Statistical analysis

Statistical analyses were carried out using SPSS version 25.0 (SPSS, Chicago, IL). The pairwise correlation analysis of the resistance against selected antibiotics, as well as qRT-PCR results, were evaluated using Pearson’s correlation coefficient. One-way ANOVA followed by Bonferroni testing were conducted for multi-variable comparison. Differences in this study were considered significant at *p* < 0.05.

### Electronic supplementary material

Below is the link to the electronic supplementary material.

Supplementary Material 1

## Data Availability

All data generated or analyzed during this study are included in this published article and its supplementary information files.

## List of abbreviations

AMUH: Alexandria Main University Hospital
AMR: antimicrobial resistance
AI-2: autoinducer-2
CAMH: cation-adjusted Mueller–Hinton
CaRV: carvacrol
CiNN: cinnamaldehyde
CLSI: Clinical Laboratory Standards Institute
DMSO: dimethyl sulfoxide
EG: eugenol
FIC: fractional inhibitory concentration
LB: Luria‐Bertani broth
MIC: minimum inhibitory concentration
MDR: multidrug-resistant
NB: nutrient broth
QS: quorum sensing
QSIs: quorum sensing inhibitors
UTIs: urinary tract infections
UPEC: uropathogenic *Escherichia coli*
