# Supplementary material for: Repurposing carvacrol, cinnamaldehyde, and eugenol as potential anti-quorum sensing agents against uropathogenic Escherichia coli isolates in Alexandria, Egypt
Source: BMC Microbiol. 2023 Oct 23;23:300. doi: 10.1186/s12866-023-03055-w (PMC10591344; doi:10.1186/s12866-023-03055-w)
Supplement: Supplementary file 1 — Supplementary Material 1 [file 12866_2023_3055_MOESM1_ESM.pdf]

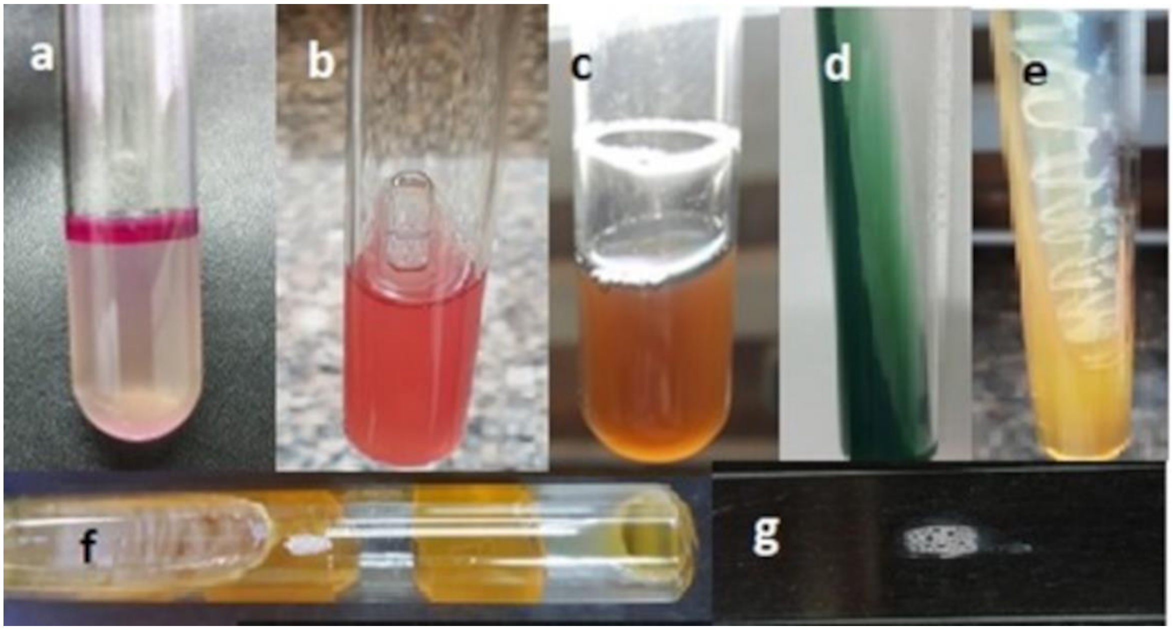

Additional file 1: Representative results of biochemical tests used for identification of *E. coli* isolates. (a) Cherry-red color in the upper organic layer indicating indole production, (b) Red color with a floating Durham tube indicating carbohydrate fermentation with gas and acid production in methyl red test, (c) Negative yellowish color in Voges-Proskauer test indicating the inability of acetoin production, (d) No change in the green color of Simmon's citrate media due to the inability to utilize citrate, (e) No observed pink coloration indicating failure to produce urease enzyme, (f) Positive triple sugar iron test showing yellow slant and yellow butt depicting fermentation of all sugars with gas production, and (g) Positive catalase test demonstrating bubble formation indicating catalase production and the breakdown of hydrogen peroxide into water and oxygen.

Additional file 2: Antibiotic resistance profiles of the tested *E. coli* clinical isolates.

Antibiotic resistance profile [a]	*E. coli* isolates	MAR index [b]
-	E32	0
DO	E34	0.067
AMC	E52	
CPM, CIP, LE	E31	0.2
CIP, DO, IPM, LE	E67	0.267
AMC, CPM, CTX, CTR, ETP	E58	0.333
CPM, CIP, DO, ETP, LE	E17	
AMC, CPM, IPM, LE, COT	E51	
AMC, CPM, CTX, CTR, DO, COT	E26	0.4
AMC, CPM, CTX, CTR, CIP, IPM, LE	E1, E30	0.467
AMC, CPM, CTX, CTR, CIP, LE, COT	E45	
AMC, CPM, CIP, DO, IPM, LE, COT	E48	
AMC, CPM, CTX, CTR, CIP, DO, IPM, LE	E5, E11	0.533
AMC, CPM, CTX, CTR, CIP, IPM, LE, COT	E15, E29, E60	
AMC, CPM, CTX, CTR, CIP, CL, IPM, LE	E61	
AMC, CPM, CIP, DO, GEN, IPM, LE, COT	E57	
AMC, CPM, CTX, CTR, CIP, ETP, IPM, LE, COT	E55	0.6
AMC, CPM, CTX, CTR, CIP, DO, ETP, IPM, LE	E6, E50, E62, E65	
AMC, CPM, CTX, CTR, CIP, ETP, GEN, IPM, LE	E10	
AMC, CPM, CTX, CTR, CIP, CL, IPM, LE, COT	E18, E28, E35	
AMC, CPM, CTX, CTR, CIP, CL, DO, IPM, LE	E16, E63	
AMC, CPM, CTX, CTR, CIP, DO, IPM, LE, COT	E9, E12, E14, E27, E39, E40, E44, E56, E66	
AMC, CPM, CTX, CTR, CIP, DO, ETP, IPM, LE, COT	E33, E38, E46	0.667
AMC, CPM, CTX, CTR, CIP, DO, ETP, IPM, LE, COT	E43	
AMC, CPM, CTX, CTR, CIP, DO, GEN, IPM, LE, COT	E2, E49, E64	
AMC, CPM, CTX, CTR, CIP, CL, DO, IPM, LE, COT	E20, E21, E22, E41	
AMC, CPM, CTX, CTR, CIP, CL, DO, GEN, IPM, LE	E24	
AMC, CPM, CTX, CTR, CIP, DO, ETP, IPM, LE, MRP, COT	E8, E19, E47	0.733
AMC, CPM, CTX, CTR, CIP, CL, DO, GEN, IPM, LE, COT	E25	
AMC, CPM, CTX, CTR, CIP, DO, ETP, GEN, IPM, LE, COT	E3, E4, E7, E59	
AK, AMC, CPM, CTX, CTR, CIP, ETP, GEN, IPM, LE, MRP, COT	E36	0.8
AMC, CPM, CTX, CTR, CIP, DO, ETP, GEN, IPM, LE, MRP, COT	E13, E37	
AMC, CPM, CTX, CTR, CIP, CL, DO, ETP, GEN, IPM, LE, COT	E42	

Additional file 2: Continued

**Antibiotic resistance profile** [a]	***E. coli*** **isolates**	**MAR index** [b]
AK, AMC, CPM, CTX, CTR, CIP, DO, ETP, GEN, IPM, LE, MRP, COT	E23, E53, E54	0.867

[a] AK: amikacin, AMC: amoxicillin/clavulanate, CPM: cefepime, CTX: cefotaxime, CTR: ceftriaxone, CIP: ciprofloxacin, CL: colistin, DO: doxycycline, ETP: ertapenem, GEN: gentamicin, IPM: imipenem, LE: levofloxacin, MRP: meropenem, TGC: tigecycline, and COT: co-trimoxazole.

[b] MAR index is calculated as the ratio between the number of antibiotics that an isolate is resistant to and the total number of antibiotics the organism is exposed to.

Additional file 3: MIC values of the phytochemicals against the tested *E. coli* clinical isolates.

***E. coli* isolates**	**MIC values (µg/mL)**		
	**Carvacrol** [a]	**Cinnamaldehyde** [a]	**Eugenol** [a]
E1	250	262.5	798.75
E2	250	262.5	798.75
E3	250	262.5	798.75
E4	250	262.5	798.75
E5	500	131.25	798.75
E6	250	262.5	798.75
E7	500	262.5	798.75
E8	500	262.5	1597.5
E9	500	131.25	798.75
E10	250	262.5	798.75
E11	250	262.5	798.75
E12	250	262.5	798.75
E13	1000	262.5	1597.5
E14	500	131.25	798.75
E15	500	131.25	798.75
E16	250	262.5	798.75
E17	500	262.5	798.75
E18	500	131.25	798.75
E19	500	262.5	1597.5
E20	500	262.5	1597.5
E21	250	131.25	798.75
E22	250	131.25	798.75
E23	500	262.5	798.75
E24	250	262.5	798.75
E25	500	262.5	798.75
E26	250	262.5	798.75
E27	250	262.5	798.75
E28	250	262.5	798.75
E29	250	131.25	798.75
E30	250	262.5	798.75
E31	250	262.5	798.75
E32	250	131.25	1597.5
E33	500	262.5	1597.5
E34	250	131.25	1597.5
E35	500	262.5	1597.5
E36	250	262.5	798.75
E37	250	131.25	798.75
E38	500	262.5	798.75

Additional file 3: Continued.

E. coli isolates	MIC values (µg/mL)		
	**Carvacrol** [a]	**Cinnamaldehyde** [a]	**Eugenol** [a]
E39	1000	262.5	798.75
E40	1000	262.5	1597.5
E41	250	131.25	798.75
E42	250	262.5	798.75
E43	500	262.5	798.75
E44	250	262.5	798.75
E45	1000	262.5	1597.5
E46	1000	262.5	1597.5
E47	500	131.25	1597.5
E48	250	262.5	798.75
E49	500	262.5	798.75
E50	500	262.5	798.75
E51	250	262.5	798.75
E52	250	262.5	1597.5
E53	500	262.5	798.75
E54	500	262.5	798.75
E55	500	262.5	798.75
E56	500	262.5	798.75
E57	250	262.5	798.75
E58	500	262.5	1597.5
E59	500	262.5	798.75
E60	250	262.5	798.75
E61	500	131.25	1597.5
E62	250	262.5	798.75
E63	500	262.5	1597.5
E64	250	262.5	798.75
E65	500	262.5	1597.5
E66	250	131.25	798.75
E67	250	262.5	798.75
ATCC 8739	250	262.5	798.75

[a] The diluent for cinnamaldehyde and eugenol was 7.5% DMSO, while for carvacrol, descending concentrations of 7.5% DMSO were applied. The MIC of DMSO against the tested isolates was determined and a concentration of 7.5% DMSO was found to allow the growth of bacteria.

Additional file 4: Fold reduction in antibiotics' MICs upon combining with 0.5X MIC of phytochemicals against selected isolates.

		E18			E35			E63		
Phyto-chemical[a]	Antibiotic[b]	MIC Antibiotic alone[C]	MIC Antibiotic +Phyto-chemical	Fold reduction in MIC of Antibiotic	MIC Antibiotic alone	MIC Antibiotic +Phyto-chemical	Fold reduction in MIC of Antibiotic	MIC Antibiotic alone	MIC Antibiotic +Phyto-chemical	Fold reduction in MIC of Antibiotic
CaRV	CIP	125	62.5	2	250	125	2	62.5	15.625	4
	CTR	16384	256	64	4096	1024	4	8192	1024	8
	CL	4	<0.125	>32	4	<0.0625	>64	4	2	2
	DO	8	0.5	16	8	1	8	128	16	8
	TGC	1	0.5	2	1	0.5	2	2	2	ND[d]
CiNN	CIP	125	125	ND	250	125	2	62.5	62.5	ND
	CTR	16384	4096	4	4096	2048	2	8192	4096	2
	CL	4	4	ND	4	2	2	4	2	2
	DO	8	0.5	16	8	8	ND	128	64	2
	TGC	1	0.5	2	1	1	ND	2	1	2
EG	CIP	125	15.625	8	250	62.5	4	62.5	7.815	8
	CTR	16384	4096	4	4096	1024	4	8192	128	64
	CL	4	4	ND	4	2	2	4	2	2
	DO	8	8	ND	8	4	2	128	8	64
	TGC	1	<0.03125	>32	1	<0.03125	>32	2	1	2

[a] CaRV: carvacrol, CiNN: cinnamaldehyde, and EG: eugenol
[b] CIP: ciprofloxacin, CTR: ceftriaxone, CL: colistin, DO: doxycycline, and TGC: tigecycline
[c] MIC values of antibiotic alone or in presence of phytochemical are measured in μg/mL
[d] ND: not detected

Additional file 5: The used primer pairs, their product size, and annealing conditions.

Gene	Sequence	Product size (bp)	Annealing temperature	Reference
csgA	F: (5'- ACTCTGACTTGACTATTACC-3')<br>R: (5'- AGATGCAGTCTGGTCAAC -3')	200	43ºC	(1)
fimA	F: (5'- TGTCCCTCAGTTCTACAGCG -3')<br>R: (5'- TCCTAACTGAACGGTTTGATC-3')	134	47ºC	(2)
fliC	F: (5'- CCAGTCTGCGCTGTCGAG -3')<br>R: (5'- CACGTTCACGCCGTTGAAC -3')	349	53ºC	(3)
gapA	F: (5'- AGTTGACCTGACCGTTCGT -3')<br>R: (5'- CACCCGCTTTAGCATCGAAC -3')	176	52ºC	(4)
luxS	F: (5'- CATACCCTGGAGCACCTGTT -3')<br>R: (5'- TGATCCTGCACTTTCAGCAC -3')	191	51ºC	(5)

References:

1. Naziri Z, Kilegolan JA, Moezzi MS, Derakhshandeh A. Biofilm formation by uropathogenic *Escherichia coli* : A complicating factor for treatment and recurrence of urinary tract infections. J Hosp Infect. 2021;117:9-16.

2. Vinothkannan R, Tamizh MM, Raj CD, Princy Sa. Fructose furoic acid ester: An effective quorum sensing inhibitor against uropathogenic *Escherichia coli*. Bioorg Chem. 2018;79:310-8.

3. Xicohtencatl-Cortes J, Cruz-Córdova A, Cázares-Domínguez V, Escalona-Venegas G, Zavala-Vega S, Arellano-Galindo J, et al. Uropathogenic *Escherichia coli* strains harboring *tosA* gene were associated to high virulence genes and a multidrug-resistant profile. Microb Pathog. 2019;134:103593.

4. Brennecke J, Kraut S, Zwadlo K, Gandi SK, Pritchard D, Templeton K, et al. High-yield extraction of *Escherichia coli* RNA from human whole blood. J Med Microbiol. 2017;66(3):301-11.

5. Kim Y-G, Lee J-H, Park S, Kim S, Lee J. Inhibition of polymicrobial biofilm formation by saw palmetto oil, lauric acid and myristic acid. Microb Biotechnol. 2022;15(2):590-602.

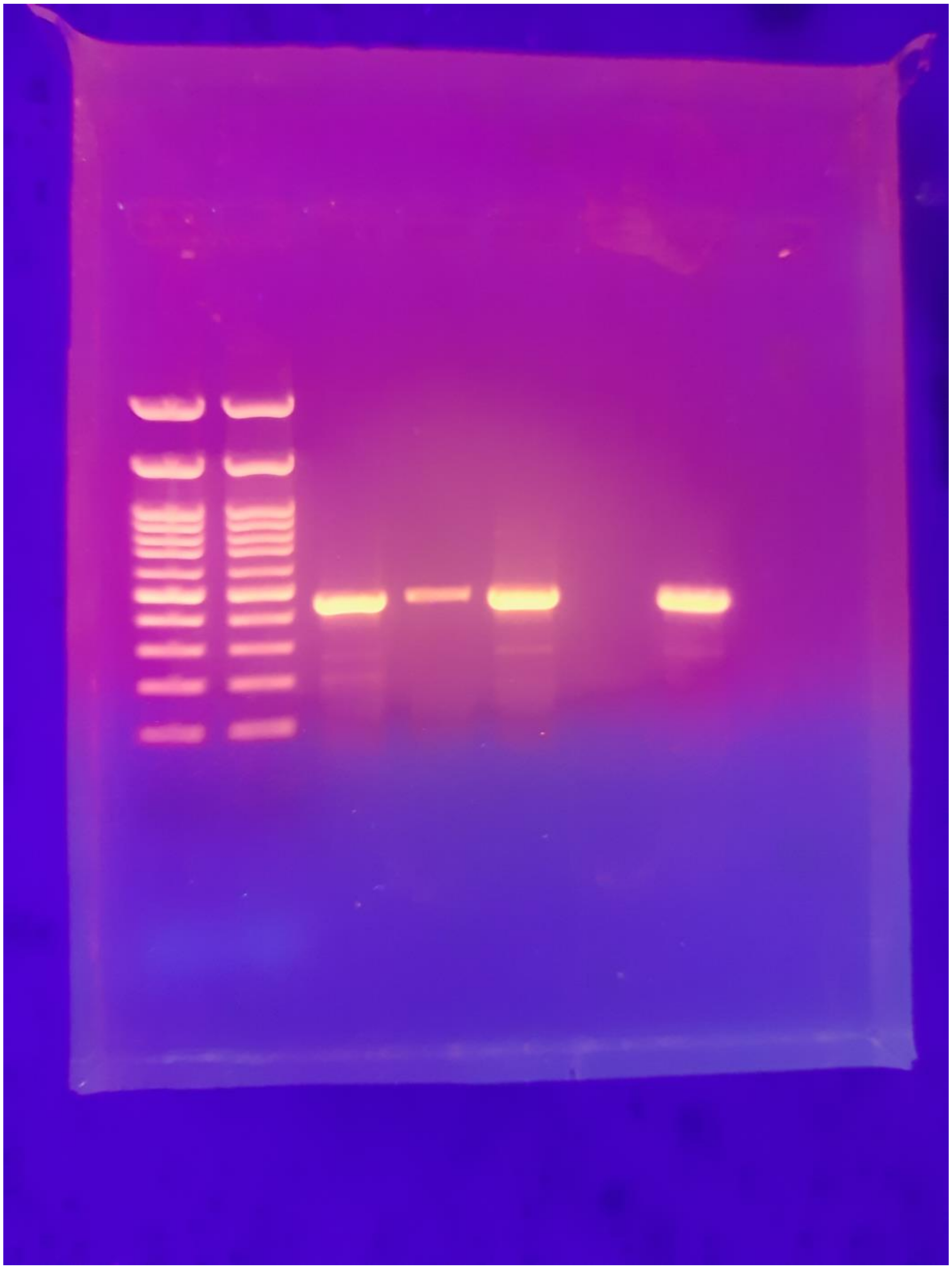

Full length uncropped unprocessed image of the agarose gel presented in the manuscript as "Figure 4": Representative agarose gel showing PCR amplification of *usp* gene in selected *E. coli* isolates. Lanes 1 and 2 correspond to a DNA molecular marker (100 bp). Lanes 3, 4, 5, and 7 show the amplicon (435 bp) corresponding to *usp* gene in E1, E2, E3, and E10 isolates, respectively. Lane 6 corresponds to the result of E5, a non-*usp*-harbouring isolate.
